# Argonin in Gonorrhea

**Published:** 1901-01

**Authors:** Nelson W. Wilson

**Affiliations:** Pathologist at the Buffalo Hospital of the Sisters of Charity


### ARGONIN IN GONORRHEA.

By NELSON W. WILSON, M. D.,
Pathologist at the Buffalo Hospital of the Sisters of Charity.

ONE often hears gonorrhea spoken of as a mere incident in the career of latter-day young men, the majority of whom appear to have not run the course of wild-oat-day pleasures unless they have been infected at least once. Some others boast of having had gonorrhea so many times that they don't mind it now at all; that it does not bother them any more than a cold in the head. We are all more or less familiar with these types; yet in spite of this popular and apparent disregard of the disease and its consequences, there is no complaint which is more difficult to properly cure and none which, when neglected or improperly treated, as it too often is, produces more distressing after-effects to the victim and those confiding females who may become their wives and eventually fall under the care of the gynecologist. The real difficulty of curing gonorrhea is evidenced by the numerous preparations put out to overcome the gonococcus, and while all may be more or less useful in various cases and at various periods in the course of the disease, there is no one which alone can be held up as an absolutely certain cure for gonorrhea. There is, however, one preparation which comes nearer to fulfilling all the requirements of an ideal treatment than any other, and this is the combination of casein and silver, known as argonin. In going over genito-urinary diseases I was struck by the great multiplicity of alleged cures and methods for gonorrhea which I found in text-books and read of in medical journals. About the only method I have not seen suggested for driving out the ever-busy gonococci is to blast them out with dynamite. You may wash, you may burn, you may drug if you will, but the biscuit-shaped bug will be with you still. I tried everything, honestly and conscientiously. I wanted to get the best possible treatment for the proper curing of gonorrhea and to that end I formed combinations of treatments. And, in order that I might be free and independent in my conclusions and utterly lacking in obligation, I purchased the material which I used. I must confess that when I took up argonin, it was with a feeling of scepticism, for I had read of really remarkable results following the use of "the sample you so kindly sent me." One report which seemed highly imaginative to me and which increased my scepticism, said that the discharge had absolutely ceased in three days. Such a gonorrheal miracle was startling to contemplate, to say the least; and with the doubt of an unbeliever I began the use of argonin. To give the results in dry Case I. and Case II. form would make prosy reading; hence they are grouped in narrative form. Altogether there were fifty-three mixed cases in which argonin was used alone or in combination.

The very first case which came under the argonin treatment was one of acute gonorrhea, with a discharge which had been free for four days. The symptoms were typical, and to avoid repetition let me add that in every case the gonococcus was found before the beginning of treatment. In this case I gave a 10 per cent. solution of argonin, to be injected twice a day with a blunt, urethral syringe of 15 cc. capacity, the injection to be retained ten minutes. In two days the patient returned with symptoms intensified and stated he was considerably worse. On inquiring how he used the syringe, he gave a demonstration. He went at his urethra as if he were chief engineer of an hydraulic pressure mining company. I reduced the strength of the injection to 5 per cent. and carefully instructed him in the use of the syringe. The second day he returned. The symptoms were slightly lessened, but he complained of the injection. I put him on alkalies, reduced the injection to 3 per cent., with orders to use it three times a day. The next visit showed marked improvement. The discharge was thin and the injection was absolutely painless. This was kept up for ten days, and then there were no painful symptoms of any character. The discharge was now scanty and very thin and colorless, and there were no gonococci found on repeated careful examinations. An astringent injection of zinc, witch hazel, hydrastis, boracic acid and water, used for a week, completed the cure. Other cases were treated variously in an experimental fashion, with argonin injections in combination with irrigations of potassium permanganate and alkalies; with methylene blue and alkalies; with methylene blue alone; with methylene blue and the permanganate irrigations, and in others the argonin injections have been the only treatment given. And the general conclusions drawn from the work done in the fifty-three argonin cases, is that argonin is by far the best thing to tie to in the treatment of gonorrhea, and if used with judgment will achieve really remarkable results. If a case is new—that is, if it is seen within forty-eight hours after the appearance of the discharge—argonin alone is a very satisfactory treatment, and in a majority of uncomplicated cases will effect a rapid and absolute cure in from four to fifteen days. In one case seen the day the discharge appeared, and in which the gonococcus was found, argonin injections alone caused the discharge to disappear on the fifth day. In this case a 2 per cent. solution was used in a 15 cc. syringe and retained ten minutes, four times a day.

In cases where the urinary symptoms were severe, the patient was given the alkaline treatment internally and the injections. I have found for urethral injections, that a 2 per cent. solution has been the most satisfactory strength, although I have used it as high as 5 per cent. in severe cases with excellent results.

The chief object to be attained, of course, is to get rid of the gonococcus as soon as possible with the least discomfort to the patient, and the sooner argonin is used the better. In some of the cases where nothing but argonin was used, chordee and painful urination disappeared in forty-eight hours. In the general run of cases the pus discharge became seromucous in from four to seven days. When this stage was reached, a single daily injection for from eighteen to twenty-one days completely cured the case. This does not mean that the discharge stopped in that time—that condition obtained much earlier—but the injections were kept up a sufficient length of time after the disappearance of every gonorrheal manifestation to insure permanency of the cure.

In subacute cases a 5 per cent. solution will be found to be much more satisfactory than the 2 per cent., which is the proper strength for acute cases.

In vulvo-vaginitis permanganate irrigations were very satisfactory, but they were slower than the argonin, which invariably produced the most brilliant results in a much shorter space of time. The strengths used were 5 per cent. and 10 per cent. for from three to five days, followed by reduction by degrees to 2 per cent. There were four cases treated and all were discharged cured on the twenty-third, twenty-ninth, thirty-first and nineteenth days respectively.

One case of ophthalmia is now under treatment with 3 per cent. argonin solution, using a medicine dropper, three times a day, and washing the lids with warm boracic acid frequently between times. Thus far eleven days have elapsed since the inauguration of the treatment, and splendid progress is being made without any involvement of the cornea. The results at present are apparently much more satisfactory than in the nitrate of silver treatment which was used in previous cases.

I do not believe that every case of gonorrhea which comes along can be absolutely cured by the use of any one agent, but with the proper internal treatment, the usual run of cases will succumb to an intelligent use of argonin as the only external agent used, and that a successful outcome will be achieved in a comparatively shorter time. It is hard to lay down a set course of treatment for gonorrhea, but argonin will be found to be the safest, the best and the most satisfactory. This conclusion is reached only after a careful study and use of all the gonorrheal remedies and by comparing them, one with the other and in all sorts of combinations, covering a period of experimentation of nearly two years.

Much depends on the preparation of the solution. If possible the surgeon should prepare it personally, unless he can send his prescription to a druggist whom he knows understands how to prepare it; and it should be dispensed in dark bottles. The most satisfactory method of preparing the solution is to first make a paste of the necessary amount of argonin powder, using hot water. To this paste add enough hot water to make the percentage solution required. This solution should be strained. Another very good method is to shake 1 part of argonin in 10 parts of cold water; add sufficient boiling water to make the desired quantity and shake until the argonin is in solution. Then strain.

Prepared either way and used properly and intelligently, argonin will produce remarkably satisfactory results in almost every case, and in some the outcome will be little short of miraculous. And when I say intelligently, I mean that the questions of hygiene, diet and alcohol must receive careful attention and the patient subjected to exact orders concerning them. With this always in mind and followed out and argonin used properly, cures will be effected which will seem as brilliant to the observer as satisfactory to the patient.

453 Niagara Street.

